# The role of particle radiotherapy in the treatment of skull base tumors

**DOI:** 10.3389/fonc.2023.1161752

**Published:** 2023-06-07

**Authors:** Alberto Iannalfi, Giulia Riva, Lucia Ciccone, Ester Orlandi

**Affiliations:** Radiation Oncology Unit, Clinical Department, National Center for Oncological Hadrontherapy (CNAO), Pavia, Italy

**Keywords:** particle radiotherapy, proton radiotherapy, carbon ion radiotherapy, skull base tumors, pediatric tumors, sellar tumors

## Abstract

The skull base is an anatomically and functionally critical area surrounded by vital structures such as the brainstem, the spinal cord, blood vessels, and cranial nerves. Due to this complexity, management of skull base tumors requires a multidisciplinary approach involving a team of specialists such as neurosurgeons, otorhinolaryngologists, radiation oncologists, endocrinologists, and medical oncologists. In the case of pediatric patients, cancer management should be performed by a team of pediatric-trained specialists. Radiation therapy may be used alone or in combination with surgery to treat skull base tumors. There are two main types of radiation therapy: photon therapy and particle therapy. Particle radiotherapy uses charged particles (protons or carbon ions) that, due to their peculiar physical properties, permit precise targeting of the tumor with minimal healthy tissue exposure. These characteristics allow for minimizing the potential long-term effects of radiation exposure in terms of neurocognitive impairments, preserving quality of life, and reducing the risk of radio-induced cancer. For these reasons, in children, adolescents, and young adults, proton therapy should be an elective option when available. In radioresistant tumors such as chordomas and sarcomas and previously irradiated recurrent tumors, particle therapy permits the delivery of high biologically effective doses with low, or however acceptable, toxicity. Carbon ion therapy has peculiar and favorable radiobiological characteristics to overcome radioresistance features. In low-grade tumors, proton therapy should be considered in challenging cases due to tumor volume and involvement of critical neural structures. However, particle radiotherapy is still relatively new, and more research is needed to fully understand its effects. Additionally, the availability of particle therapy is limited as it requires specialized equipment and expertise. The purpose of this manuscript is to review the available literature regarding the role of particle radiotherapy in the treatment of skull base tumors.

## Introduction

The skull base is an anatomically complex and functionally critical area. Because of their anatomical location, the management of skull base tumors is challenging for both neurosurgeons and radiation oncologists.

Surgery is often the first step in therapeutic management to obtain pathologic sampling, improvement of symptoms, and cytologic reduction. Due to the proximity of critical vasculo-nervous structures, total removal of the tumor is often not possible or could be achieved at the price of potentially life-threatening complications (1, 2).

Maximum safe surgical resection, usually followed by radiation therapy (RT), represents the standard of care for many skull base tumor histologies, both malignant and benign.

Improvements in RT technology, such as intensity-modulated radiotherapy (IMRT) or volumetric arc therapy (VMAT), have allowed precise delivery of RT doses to skull base lesions.

However, due to the proximity to some organs at risk (OARs), such as the brainstem and the optic pathways, and the need to deliver very high doses (even over about 70 Gy) for radioresistant histologies, RT with photons may not be sufficient to obtain good control of disease without side effects.

Particle radiotherapy (PRT) using protons or heavy ions is probably currently the most advanced form of RT and offers new opportunities for improving cancer care and research.

**Figure 1:**
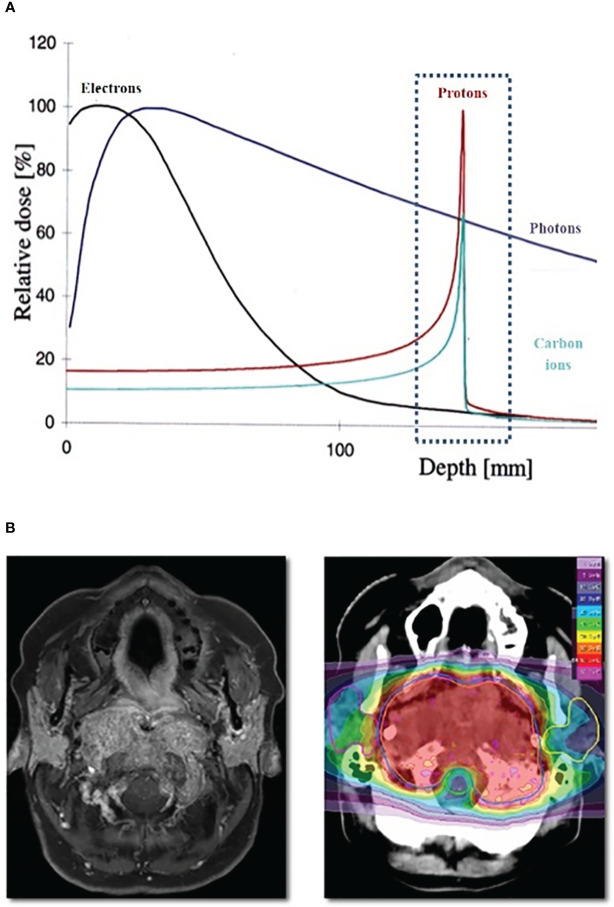
**(A)** Bragg Peak Curve Plot; **(B)** Example of particle radiotheraphy plan for skull base tumor.

Protons and heavy ions, such as carbon ions, can potentially improve dose sparing of normal tissues through the exploitation of the Bragg peak phenomenon, resulting in an increase in energy deposition with penetration depth up to a sharp maximum followed by a rapid decrease at the end of the penetration range (3). These features permit more precise and conformal dose localization to the target compared with conventional photon RT **(** **Figure 1**).

Moreover, considering carbon ions, other biological advantages are provided in addition to the improved physical dose distribution, owing to the high relative biological effectiveness (RBE) of radiation with high linear energy transfer (LET) (4).

RBE is the parameter that expresses quantitatively the biological effect of PRT, which is the ratio between the reference photon radiation and the PRT that produces the same biological effect.

Because carbon ion beams have a high LET, they can create clusters of DNA damage that cannot be repaired. Carbon ions are more effective than protons and photons for the treatment of hypoxic cells, with fewer variations in radiosensitivity related to the cell cycle.

Currently, according to the Particle Therapy Co-Operative Group (PTCOG) website, there are 115 particle therapy facilities clinically active: 101 proton centers, eight carbon ion centers, and six centers with both carbon ions and protons (5).

This review summarizes published literature and assesses the present status regarding the role of proton (PT) and carbon ion (CIRT) therapy in skull base tumors.

## Chordomas

Chordomas are rare primary bone tumors arising from notochord remnants, with an incidence of 0.8–1 case per 1 million population/year (6). These tumors arise mostly in the axial skeleton, with the midline clivus involved in approximately one-third of cases.

Currently, the World Health Organization (WHO) histologically defines three types of chordoma: conventional chordoma, dedifferentiated chordoma, and poorly differentiated chordoma (7). Chordoma is immunopositive for epithelial markers such as cytokeratins (CKs) and endothelial membrane antigen (EMA) and can also be positive for S-100 and vimentin (8). Brachyury was recognized as the diagnostic hallmark for chordoma staining to discriminate chordomas from histological entities with similar morphological characteristics (7). In poorly differentiated chordomas, tumor cells are positive for broad-spectrum CKs and brachyury; they show loss of SMARCB1/INI1, and the S100 protein is rarely expressed (9).

Chordomas are locally aggressive and invasive and generally slow-growing; therefore, they are often clinically silent until the late stages of the disease. Clinically, patients mostly present with headache and cranial nerve deficits (especially diplopia, vision impairment, and trigeminal neuralgia), sensorimotor deficits, pituitary dysfunction, and hydrocephalus (10). Metastases are rare at the time of diagnosis and can occur in the lung, liver, bone, lymph nodes, and other sites, but the prognosis is more related to the local aggressiveness of chordoma than to its potential to metastasize.

The large tumor burden at the time of diagnosis and the surrounding critical structures, such as the brainstem, cavernous sinus, and optic apparatus, could often preclude a gross total resection (GTR). Surgery should aim towards maximum tumor resection combined with preservation of neurological function and quality of life, decompressing the brainstem and optic pathway, and reducing the volume of disease to enhance the effectiveness of subsequent RT (11–13).

Due to the low radiosensitivity of chordomas, different studies have reported that a dose escalation to at least 70 Gy is needed to improve tumor control rate, even though these doses are often difficult to achieve with conventional photons due to directly adjacent vital structures (14, 15).

Advances in RT technology with the introduction of PRT have led to higher doses of radiation delivered to the target volume with minimal injury to the surrounding tissue and improved radiobiological effects (7).

Both PT and CIRT have been successfully used in the treatment of skull base chordoma, and good results have been reported with limited severe acute and late toxicity and a high probability of local control (LC). Because protons have a longer treatment history and have been applied in multiple centers over the past 25 years, their series are larger than carbon ions (16–23) **(** **Table 1** **)**.

**Table 1:** Patients and treatment description of chordomas irradiated with proton or carbon ion (selected series).

Study	Particle	Patients (number)	Follow-up (months)	RT Dose (GyRBE)	LC (%)	OS (%)	Severe late toxicity
Hug, 1999 (16)	P	33	33 (median)	TD: 65–79 Dpf: 1.8–2	5-y: 59	5-y: 79	7%
Munzenrider, 1999 (17)	P + ph	169	41 (median)	TD: 66–83 Dpf: 1.8–1.9	5-y: 73 10-y: 54	5-y: 80 10-y: 54	Disaggregated data not reported or limited cohort followed-up for toxicity outcomes
Uhl, 2014 (18)	C	155	72 (median)	TD: 60 (median) Dpf: 3	5-y: 72	5-y: 85	0%
Weber, 2016 (19)	P	151	50 (mean)	TD: 72.5 (mean) Dpf: 1.8–2	7-y: 70.9	7-y: 72.9	8%
Fung, 2018 (20)	P + ph	106	61 (mean)	TD: 8.4–73.8 Dpf: 1.8	5-y: 75	5-y: 88	7%
Koto, 2019 (21)	C	34	108 (median)	TD: 60.8 (median) DpF: 3.8 (median)	5-y: 77	5-y: 93	11%
Iannalfi, 2020 (22)	P	135	49 (median) whole series	TD: 74 (median) Dpf: 1.8–2	5-y: 84	5-y: 83	12% (2% expected for tumor very close to optic nerve and/or pre-existing severe deficit). No G3 brain necrosis.
Iannalfi, 2020 (22)	C	65	49 (median) whole series	TD: 70,4 Dpf: 4.4	5-y: 71	5-y: 82	12% (2% expected for tumor very close to optic nerve and/or pre-existing severe deficit). No G3 brain necrosis.
Mattke, 2022 (23)	P	36	36 (median)	TD: 74 (median) Dpf: 1.8-2	5-y: 61	5-y: 92	13% (cumulative rates of brain injury) G3 toxicity reported, but disaggregated data for G3 not reported
Mattke, 2022 (23)	C	111	52 (median)	TD: 66	5-y: 65	5-y: 83	13% (cumulative rates of brain injury) G3 toxicity reported, but disaggregated data for G3 not reported

P, proton; C, carbon; RT, radiotherapy; TD, total dose; Dpf, dose per fraction; LC, local control; y, years; OS, overall survival.

Five-year LC ranged between 59% and 84%, whereas 5-year overall survival (OS) rates ranged from 72% to 83%. The largest published study reported 10-year LC and OS rates of 54% and 75%, respectively (18).

Some prognostic factors for LC have consistently been reported to be of predictive value, whereas others have only been sporadically discussed. Regarding the influence of residual tumor volume after surgery, many series have reported the residual tumor volume to be a prognostic factor for LC, with different cut-off points ranging from 10 cc to 75 cc (18–22, 24).

Furthermore, the presence of low-dose regions and dose inhomogeneity within the gross tumor volume (GTV) is a primary reason for local recurrence. The underdosing of a tumor’s portion may increase the risk of local recurrence, but on the other hand, some portions of the target are underdosed to meet constraints on critical normal structures; this critical situation is intrinsically due to the occurrence of disease in very close proximity to or involving brainstem or optic pathways (22). The prognostic factors emphasize the importance of the combination strategy of maximally safe resection followed by PRT, which permits high biologically effective doses. In the event that that the surgeon determines that a maximally safe resection is not feasible, a debulking “space” surgery that creates distance between tumor and organ at risk should be considered to favor the delivery of a high RT dose in the most optimal way by PRT. In this perspective, the sharing of combined treatment planning strategies between the surgeon and radiation oncology is crucial to obtaining the most favorable clinical outcomes within the framework of the network, which includes highly specialized centers for the management of skull base tumors.

Although Munzenrider et al. raised the issue of female patients having decreased local control rates (17), this data was not confirmed by other series, and, according to a recent retrospective analysis of 238 patients with skull base chordomas, sex was not found to have a predictive value (24).

Moreover, except for Jahangiri and colleagues, who identified tumor localization in the middle and lower third of the clivus as other risk factors for recurrence, no relation between the site of residual tumor and LC was reported in other series (25).

In primary PRT, the target volume delineation should be primarily based on the concept of risk-based volumes, considering preoperative disease extension, potential dissemination ways, data emerging from a detailed surgical report (as dural infiltration), and post-operative changes (12, 22).

Loco-regional relapse is a relatively common pattern of recurrence following initial treatment of chordoma patients and includes progression of the treated primary tumor, lesions recurring near surgical margins, or lesions developing because of iatrogenic seeding along a biopsy or surgical tract (26, 27). Salvage treatment choices represent a major clinical challenge and can include surgery and/or RT, balancing morbidity and expected disease control.

The choice of the best treatment strategy between surgery alone, surgery plus RT, and RT alone must be based on an individual case evaluation. Potential eligible patients for a complete surgical re-intervention are patients presenting isolated disease, a long disease-free interval, good performance status, and a reasonable likelihood of acceptable morbidity (26).

Uhl et al. (27) reported outcomes regarding reirradiation (re-RT) with carbon ions performed on 25 patients with locally recurrent skull base chordoma (n = 20) or chondrosarcoma (n = 5). Fourteen of the patients underwent PRT (CIRT/PT) as previous RT, with a median dose of 60 GyE (range: 42–72 GyE), while 11 of them had photon therapy with a median dose of 66 Gy. The median applied total dose of re-RT with carbon ions was 51 GyE (range: 45–60 GyE) in five to six fractions of 3 GyE per week, and it was reported as correspondence to a median equivalent dose of 63.8 GyE (range: 56.2–75 GyE) calculated for a fraction dose of 2 Gy (EQD2 Gy) and an alpha/beta ratio of 2. The 2-year local progression-free survival (PFS) probability was 79.3%. Five cases of recurrence occurred in chordomas, but only one in chondrosarcomas. A planning tumor volume (PTV) of <100 ml or a total dose of >51 GyE was correlated with an improved LC rate. Low acute toxicity was described: one patient developed grade (G) 2 mucositis during therapy, while three patients had hypoacusis related to a new onset of temporary middle ear effusion (G2). Furthermore, five patients developed an asymptomatic temporal lobe reaction after treatment without the need for surgical intervention (G1). Only one patient had a G3 osteoradionecrosis in the treatment area 1 month after irradiation, which required surgery. In 84% (21/25) of patients, the tumor-associated symptoms were stable or had decreased after therapy (27).

In the case of recurrent disease after previous RT, a re-RT can be indicated only in the following situations: re-RT can be delivered without exceeding the estimated dose tolerance limits on OARs, and appropriate dose coverage of target volumes can be obtained. Conversely, other treatment strategies should be preferred. Currently, the cumulative dose tolerance for the most critical OARs and the degree of recovery of healthy tissue receiving radiation dose after the first course of RT and its potentially protective role are still widely preliminary and very difficult to estimate (26, 28).

In the case where a complete macroscopic resection of a recurrent lesion cannot be likely obtained and proximity to critical structures does not permit adequate RT coverage of target volumes, debulking “space” surgery may be an adequate solution to create a distance between the critical structures and the residual recurrent tumor, thereby allowing delivery of a tolerable radiation dose (26).

The radiation oncologist must be develop the radiation plans based on an accurate reconstruction of the previous RT dose distribution and taking into account expected morbidity of second radiation. If a re-RT can be delivered without exceeding the estimated dose constraints on OARs, the patient should be treated with the same intent and approach as a RT naïve recurrence. When this goal is not achievable, sufficient data are not available to recommend an optimal dose and fractionation scheme for radiation in this setting and radiation oncologists should develop a treatment plan for obtaining the best balance between the higher RT dose with adequate target volume coverage and respecting dose constraints (26).

In cases of tumor seeding in the surgical pathway, the site of recurrence is often “out of field” regarding the previously irradiated volume, and the relapsed site can be adequately and easily treated by RT at higher curative doses. Low-dose re-RT with palliative intent can be appropriate in selected cases, but only if it can be performed with a negligible risk of toxicity. The use of high-LET radiation, such as CIRT, may be estimated as a more effective option against the radioresistant clones that may have been selected by the first treatment (26).

Due to the lack of sufficient data to assess dose tolerance in relation to toxicity in the re-RT of chordomas, the dose/fractionation schemes for re-RT cases have remained heterogeneous and based primarily on qualitative evaluation of the prior treatment plan. Caution is warranted in re-irradiating the carotid artery because of life-threatening complications such as carotid blowout (CBO) syndrome that have been reported in patients treated with re-RT for head and neck cancer (28).

In a recently published review, it emerged that a higher risk of CBO is likely awaited when a higher cumulative dose than 120 Gy is delivered to the carotid artery in received RT courses (28). The risk of CBO represents consequently a critical concern, often limiting the indication of the re-RT option for skull base chordomas, considering the high dose required in radiation treatment of chordomas in both primary RT and re-RT settings (26).

When re-RT, especially with carbon ions, represents the required main salvage treatment option, in the case of high-risk patients for threatened or impending CBO in the current practice, the multidisciplinary team should carefully evaluate the best preventive strategy for CBO (surgical ligation, stenting, or occlusion), similarly to how much is suggested for head and neck tumors (28).

In RT treatment of chordomas located in the lower third of the skull base and extended to the cervical spine, metal implants (e.g., for cranio-cervical stabilization) can make RT delivery more complicated by creating artifacts in radiological imaging and interfering with precise delineation of target and OAR, especially in the spinal canal. Furthermore, these artifacts affect the range calculation for PRT, determining additional uncertainty in the delivered dose (12, 26). Theoretically, the presence of metal implants should be considered a critical factor in deciding not to deliver curative RT or in deciding to deliver it with photons, which are less sensitive to artifacts compared to particles.

Especially in the setting of patients with newly diagnosed skull base chordomas invading the cranio-spinal junction and extending to the cervical spine, a multidisciplinary assessment involving a surgeon and radiation oncologist is mandatory to plan a better combined strategy. In many cases, this problem can be resolved in the current practice by sharing the best geometric arrangement of craniocervical metal implants and screw fixation to obtain stabilization and be compatible with the particle beam geometry assessment estimated based on disease extension on pre-surgery imaging. In other cases, the shared decision by surgeons and radiation oncologists to postpone stabilization after PRT can represent the best option.

In the case of treatment of recurrent disease with metal implants previously positioned, especially if a debulking or separating surgery is planned, the possibility of modifying, removing, or substituting metal implants with non-metal implant devices (carbon fiber devices) should be considered to enable radiation with potentially curative intent; however, this represents an appropriate choice only in very well selected cases after an accurate multidisciplinary evaluation (26). It is important to underline that in craniocervical junctions, in current practice, the required curvature of bars is very often not compatible with the availability of carbon fibers (or other non-metal based) devices.

## Chondrosarcomas and other sarcomas

Chondrosarcomas are a heterogeneous group of slow-growing neoplasms originating from cartilage-producing cells in enchondral ossification areas, with an incidence of 0.2 per 100,000 cases (29). At the base of the skull, common sites of involvement are usually represented by the temporo-occipital junction, parasellar area, spheno-ethmoidal complex, and clivus.

WHO grading of chondrosarcomas is essential and useful in predicting histological behavior. Chondrosarcomas are divided into three grades based upon their histopathology: grade I, considered to be low-grade and usually indolent with minimal malignant potential regardless of their location and presentation; grade II; and grade III (30). A fourth group considered grade IV, makes up 10% of all chondrosarcomas and, by definition, is a high-grade neoplasm with an inferior prognosis (31).

Histological subtypes include the following: classical/conventional (85% of all chondrosarcomas), mesenchymal, clear cell, and dedifferentiated (10, 31).

Skull base chondrosarcomas are slow-growing tumors that gradually progress at the base of the skull structures from abutting or encasing them to subsequently invading critical organs. Most patients are asymptomatic or develop symptoms at a later stage of the disease as a result of infiltration and compression of the surrounding neural structures (headache, diplopia secondary to abducens nerve palsy, lower cranial nerve deficits) (32).

The dominant failure pattern after treatment for a skull base chondrosarcoma is local recurrence, and surgery is the cornerstone of the primary management of this disease. However en bloc resection/GTR with sufficient surgical margin is universally challenging due to the complexity of the anatomy. In 2009, a systematic literature review demonstrated a significant reduction in the 5-year rate of local recurrence from 44% after surgery alone to 9% after RT (29).

However, to achieve adequate LC, high radiation doses are necessary due to relatively high radioresistance. Given the need for high doses and the sparing of the OARs, PRT has been used in the treatment of skull base chondrosarcomas to accomplish this goal.

The review by Amichetti et al. reported 5-year and 10-year LC rates after PRT in patients with chondrosarcomas of the skull base of 75% to 99% and 98%, respectively (33).

In **Table 2**, clinical outcomes of the largest series of skull base chondrosarcoma treated with PRT are summarized (16, 34–36).

**Table 2:** Patients and treatment description of chondrosarcomas irradiated with proton or carbon ion (selected series).

Study	Particle	Patients (number)	Follow-up (months)	RT Dose (GyRBE)	LC (%)	OS (%)	Severe late toxicity
Hug, 1999 (16)	P	25	33 (median)	TD: 69.3 (mean) Dpf: 1.8	5-y: 75	5-y: 100	7%
Weber, 2016 (34)	P	77	69.2 (mean)	TD: 70.0 (mean) Dpf: 1.8–2	8-y: 89.7	8-y: 93.5	8%
Mattke, 2018 (35)	P	22	30.7 (median)	70 (median)	4-y: 100	4-y: 100	0%
Mattke, 2018 (35)	C	79	43.7 (median)	60 (median)	4-y: 90.5	4-y: 92.9	0%
Riva, 2021 (36)	P	32	31 (median)	TD: 74 (median) Dpf: 2	3 y LC: 100%	–	6%
Riva, 2021 (36)	C	16	66 (median)	70.4 Dpf: 4.4	3 y LC: 94%	–	12%

P, proton; C, carbon, RT, radiotherapy; TD, total dose; Dpf, dose per fraction; LC, local control; y, years; OS, overall survival.

Chondrosarcoma is perhaps the most common histotype of sarcoma starting at the skull base, but it is not the only one. Literature on the use of PRT for the treatment of the base of skull sarcomas other than chordoma and chondrosarcoma, which are usually more aggressive, is scarce (37, 38).

Few data concerning other types of skull base sarcomas are reported in case series or in inclusive studies of different sarcomatous histologies, such as the study by Yang and colleagues published in 2020. In this study, the authors reported the clinical outcomes of 62 patients with skull base bone or soft-tissue sarcomas (chordoma excluded) treated with PRT (both proton and carbon ion) as primary RT or re-RT for relapse (38).

Among the 45 radiation-naïve patients in this study, 2-y PFS and OS were 62.9% and 80.2%, respectively (38).

However, for a rare condition such as skull base sarcoma, it will be difficult to perform prospective randomized trials concerning PRT for each histological subtype of the disease because patients’ treatment in terms of surgery and chemotherapy varies substantially.

## Meningiomas

Meningiomas are the most common primary intracranial tumor, with an incidence rate of 37.6% (39).

The WHO 2021 classifies meningiomas into three different histopathological types: grade I (benign), which has a low recurrence rate and accounts for 80% of cases; grade II (atypical), which comprises 20%–30% of patients and has a recurrence rate of 30%–40%; and grade III (anaplastic), which is found in 1%–2% of patients and almost surely recurs (40).

The diagnosis of meningioma is most common in middle and old age. The frequency increases with age, and women are twice as likely to be diagnosed as men (39, 41, 42).

The occurrence risk of a meningioma is linked to previous cranial exposure to ionizing radiation and previous brain RT in childhood. Furthermore, the risk is associated with a genetic condition called type 2 neurofibromatosis (NF2). In fact, NF2 patients are more likely to develop WHO 2–3 or multiple meningiomas (39).

Finally, growing data suggest an association between the prolonged exposure of women to endogenous or exogenous sex hormones and meningioma. An association with breast cancer and a higher incidence in reproductive age (increasing during pregnancy and decreasing after delivery) and in menopause is reported. Progesterone receptor expression may be involved in the occurrence of meningiomas (43).

With the increase in neuroimaging availability, incidental meningioma diagnoses have increased. The 1% of the general population that undergoes a brain magnetic resonance imaging (MRI) presents an incidental meningioma (39).

Surgically accessible meningiomas that can be safely removed have indications for surgical resection. The cornerstone of symptomatic or growing meningiomas is maximal surgical resection, minimizing morbidity and preserving neurological functions. However, as happens with skull base tumors located close to the cavernous sinus, total removal is rarely achieved without a planned subtotal resection. Incomplete surgical removal is associated with an increased risk of progression. Afterward, according to grade, residual meningioma can be monitored or treated with postoperative RT. The interval from surgery to progression can be long, and the timing of RT after incomplete surgery or when meningioma relapses remains questioned (2).

External beam RT improves LC, and new advanced radiation techniques can provide excellent target dose coverage, precise target localization, and accurate dose delivery. Photon-based RT is usually recommended as adjuvant therapy or as the primary treatment for meningioma. Several RT techniques have been developed: IMRT, VMAT, and stereotactic irradiation modalities (i.e., Gamma Knife, CyberKnife) (39, 44).

PRT is an option in meningioma management as an alternative to photon RT. PT is the most common PRT used in clinical practice (40, 42). Another option is represented by CIRT, whose use is reserved especially for re-RT after disease progression (41).

PT has a radiobiological superior advantage over photon RT due to the capability to deposit most of the particle energy at the end of their trajectory with a very little exit dose beyond the target, sparing surrounding healthy tissues. Furthermore, PRT is characterized by a RBE equal to 1.1 and 1.5–3.0 for proton and ion-carbon, respectively (45).

Consequently, for patients with potentially long-term survival, PT may be proposed for skull base meningiomas, especially in cases of complex shapes and larger volumes.

Moreover, a better profile of dose distribution decreases the risk of treatment-related side effects (i.e., radionecrosis and neurocognitive impairment) and the risk of potential radiation-induced secondary malignancy (2). In the dedicated paragraph below, selection criteria for PRT in low-grade skull base tumors are discussed. In a smaller portion of the patients, the meningiomas with skull base location present higher-grade types (WHO II–III), which required a higher dose level. For this reason, the achievement of the most favorable ratio between optimal coverage of treatment volume with a therapeutic higher dose and the sparing of tolerance dose to critical structures can further represent a critical advantage of PT in the treatment of higher-grade skull base meningiomas, especially in cases with the closest proximity of tumors with brainstem and/or optic pathways.

PT is successfully utilized for meningioma (skull base and other localizations) treatment, with both a good achievement of LC and a few reports of acute and severe toxicities. Characteristics of the principal studies of PT for meningioma are summarized in **Table 3** (46–54).

**Table 3:** Patients and treatment description of meningiomas irradiated with proton or carbon ion (selected series).

Study	Site	WHO grade	Particle	Patient (number)	Follow up (Month)	RT dose (GyRBE)	LC (%)	OS (%)	Toxicity
Gudjonsson, 1999 (46)	Skull base	15 (G1) 4 (Unknown)	P	19	36 (at least)	TD: 24 DpF: 6	3-y: 100	/	No severe toxicity
Vernimmen, 2001 (47)	Skull base	23 (G1)	P	23	40 (mean)	TD: 54-61.1 Dpf: 16-27	5-y: 88	/	Late toxicity (any grade): 11%
Weber,2004 (48)	Skull base + Other sites	11 (G1) 2 (G2)	P	13	34 (median)	TD: 56 (median) Dpf: 1.8-2	3-y: 100	3-y: 84	Late toxicity (any grade): 19%
Halasz, 2011 (49)	Skull base + Other sites	50 (G1)	P	50	32 (median)	TD: 13 DpF: 13	3-y: 94	/	/
Weber, 2012 (50)	Skull base + Other sites	23 (G1) 9 (G2) 2 (G3) 5 (Unknown)	P	39	54.8 (median)	TD: 56 (median) Dpf: 1.8–2	5-y: 84.6	5-y: 82	Late toxicity (any grade): 41% Severe late toxicity: 13%
Combs, 2013 (51)	Skull base	71 (G1) 36 (G2-3)	P ± C boost	107	12 (median)	TD P: 52.2–57.6 TD C: 18	LC at the end of FUP WHO G1: 100 2-y WHO G2–3: 33%	3-y: 100	/
Murray, 2017 (52)	Skull base + Other sites	61 (G1) 35 (G2–3)	P	96	56.9 (median)	TD WHO G1: 54 (median) TD WHO G2-3: 62 Dpf: 1.8–2	5-y WHO G1: 95 5-y WHO G2: 69	5-y WHO G1: 92 5-y WHO G2: 80	Late toxicity (any grade): 45% Severe late toxicity: 10%
Vlachogiannis,2017 (53)	Skull base + Other sites	170 (G1)	P	170	84	TD: 14–46 Dpf: 3–8	5-y: 93	/	Late toxicity (any grade): 9%
El Shafie, 2018 (54)	Skull base	60 (G1) 7 (G2) 1 (G3) 42 (Unknown)	P +/- C boost	110	46.8 (median)	TD P: 54 TD P+C: 50 P + 18 C	5-y: 96.6	5-y: 96.2	Severe late toxicity: 3.6%

WHO, World Health Organization; P, proton; C, carbon; RT, radiotherapy; TD, total dose; Dpf, dose per fraction; LC, local control, y, years, OS, overall survival.

Meningiomas often recur over time, regardless of the initial extent of surgery, and repeating surgery with/without the use of adjuvant therapeutic options may be necessary. PT may be a treatment option when surgery is not feasible. In fact, due to its higher RBE, it is feasible to treat more radioresistant diseases, such as a recurrence of pre-irradiated meningioma, with a lower burden of side effects.

Champeaux-Depond et al. reviewed 193 cases of recurrence or progression of meningioma that underwent PT (55).

Five-year PFS was 71.5% (95% CI 64.4–79.4), 55.6% (95% CI 32.5–95), and 35.6% (95% CI 12.8–98.9) for WHO G1, G2, and G3 meningiomas, respectively. Five-year OS rates were 93% (95% CI 88.7–97.4), 76.4% (95% CI 51.4–100), and 44.4% (95% CI 16.7–100) for WHO G1, G2, and G3 meningiomas, respectively (55). Recurrences after RT in patients with meningiomas generally represent a very challenging clinical situation: prior RT has often completely saturated the margin of radiation tolerance of critical organs, and for this reason any additional RT must be performed using highly advanced RT modalities. Skull base location represents a very critical feature, which further contributes to the high degree of difficulty in performing effective re-RT. In terms of treatment alternatives, the risk of neurosurgical intervention can be associated with high rates of treatment-related sequelae.

El Shafie et al. (56) published the results of 42 patients treated with PRT for recurrent intracranial meningioma after previous irradiation. The location was the skull base in 73.8% of patients. Concerning dose received in previous RT, the median dose was 52.9 Gy (range 12.1–62.4 Gy) for IMRT (n = 16 patients), while the median dose for 3-dimensional conformal RT (n = 16 patients) was 54 Gy (range 50.5–55.8 Gy); seven patients received stereotactic radiosurgery (SRS) at a median dose of 12.1 Gy (range 12.0–17.0 Gy), and one patient received fractionated stereotactic RT (FSRT) at a cumulative dose of 58.8 Gy. One patient had previously received radiopeptide therapy with Y-90 DOTATATE at 4.39 GBq, corresponding to an approximated local dose of 10 Gy, whereas another patient previously received previous CIRT due to tumor progression. The patients were treated with PT in 19% of cases (n = 8) and CIRT in 81% of cases (n = 34). The median total dose of PRT was 51 Gy (RBE) [range 15–60 Gy (RBE)]. Four patients received bimodal treatment with a carbon ion boost and a photon base plan: 15 Gy (RBE) (n = 1) or 18 Gy (RBE) (n = 3), applied after 50–52 Gy of photon irradiation. For CIRT, most commonly, a dose per fraction of 3 Gy (RBE) was applied, as well as a dose per fraction of 3.3 Gy in one case. For PT, smaller doses per fraction, such as 1.8 Gy (RBE) or 2 Gy (RBE), were used. Different fractional schemes of PRT were applied depending on the previous treatment dosimetry, and the goal was to deliver a dose upward of 50 Gy (RBE) for WHO-1 tumors and upward of 54 Gy (RBE) for higher-grade tumors. The PFS after 12 months accounted for 71% and 56.5% after 24 months, and the OS was 89.6 and 71.4%, respectively. Histology impacted PFS significantly for high WHO G2/G3 tumors; the median PFS was 25.7 months, while the median PFS was not reached for WHO 1 tumors due to a limited number of events. No significant difference in PFS could be detected between WHO G2 and G3 meningiomas. Notably, it is relevant that the tumor volume treated was large: the mean GTV was 51.3 ml, while the median GTV was 18.1 cc. The OS after re-RT was 89.6% after 12 months and 71.4% after 24 months, with a median OS of 61 months (95% CI 34.2–87.7). The WHO grading had a relevant effect, as the median OS for low-risk patients was not reached, whereas for high-risk patients it was 45.5 months. Treatment was performed safely without interruption, and no G4 or G5 toxicities were observed. In total, three patients developed radiation necrosis; two required surgeries (G3), and one was treated with corticosteroid administration (G1) (56).

Imber et al. (57) reported a review of 16 patients who received PT re-RT for recurrent meningiomas. The location was the skull base in 69%. At diagnosis, 44%, 50%, and 6% of patients presented WHO G1, G2, and G3 tumors, respectively. The median dose received with prior RT was a median of 54 Gy (range 13–65.5). The median time between the prior RT and the PT re-RT was 5.8 years (range 0.7–18.7). The median PT dose was 60 Gy (RBE) (range 30–66.6), and the median PTV was 76 cm^3^ (range 8–249). The median follow-up was 18.8 months. At the last follow-up (range 1.2–41.5 months), 44% of intracranial recurrences and 19% of disease-related deaths were found. The median cohort PFS was 22.6 months, with 1- and 2-year PFS of 80% and 43%, respectively. Median OS was not achieved, with 1- and 2-year OS of 94% and 73%, respectively; all deaths were attributed to being related to meningioma. Patients with initially WHO G1 tumors presented significantly improved PFS versus higher grades with 1- and 2-year PFS estimates of 100% versus 71% and 75% versus 29%, respectively. Longer intervals between prior RT and PT also predicted improved PFS (P = .03) and OS (P = .049). Overall, the late G ≥3 toxicity rate was five out of 16 patients. The most common post-treatment complication was new or worsening hydrocephalus in three patients. A review of the imaging acquired to plan PT re-RT suggested that all three patients had some degree of baseline radiological evidence of ventriculomegaly. Two patients (13%) developed radionecrosis at 6 and 16 months after PT; only one was symptomatic (57).

## Craniopharyngiomas

Craniopharyngiomas (CPs) are rare, histopathologically neuroepithelial tumors arising from the embryological remnants of the primitive craniopharyngeal duct, or Rathke’s pouch. Despite their histopathologically low-grade classification, these patients frequently experience profound disabilities that affect their quality of life and instrumental daily activities. CPs present two classically distinct subtypes in adults: adamantinomatous (ACP) and papillary (PCP). In children, ACP is nearly total. The overall incidence of CPs is reported as 0.13–0.16 in 100,000, constituting 5%–10% of pediatric and 1%–4% of adult brain tumors, respectively (58).

The age distribution was bimodal, with one peak in 5- to 9-year-olds and another in 55- to 69-year-olds. Compared with ACP, PCP only represents 5.5% of the histologically diagnosed CPs in 0- to 29-year-olds (58).

Primarily in children affected by CPs, several studies supported the idea that the pre-operative hypothalamic involvement should address treatment strategy towards a conservative surgical approach followed by RT aimed at hypothalamic damage sparing (59–64).

In a recent consensus paper regarding surgical management of CPs in adult patients published by EANS (European Association of Neurosurgical Societies), it was recommended performing a GTR when there is no infiltration of the hypothalamus, while performing subtotal resection (STR) coupled with adjuvant RT when hypothalamic infiltration is confirmed (hypothalamic-sparing resection). Furthermore, the authors recommended the use of traditional endonasal trans-sphenoidal approaches for purely intrasellar CPs and suggested performing an expanded endonasal trans-sphenoidal approach as a first-line surgical approach for midline and retro-chiasmatic CPs without lateral extension (65).

The endoscopic endonasal approach (EEA) series can achieve high rates of GTR (68.9%) and satisfactory clinical outcomes: 64.3%–78.9% GTR rates for purely infra-diaphragmatic CPs and 66.3% GTR rates in lesions involving the supradiaphragmatic space (66). The main advantage of endoscopic EEA has been observed in more complex supradiaphragmatic lesions, which can be treated effectively and safely with this approach (67). When both approaches are feasible, the endoscopic endonasal approach has been found to be significantly associated with better surgical outcomes compared with transcranial approaches in terms of GTR rates and visual outcomes. Furthermore, favorable results for EEA have been related, though not significantly, to complications such as panhypopituitarism and diabetes insipidus. No significant rates of meningitis have been recorded between the two surgical approaches, although TCA showed a significantly lower risk of CSF leakage (68).

Furthermore, the endoscopic endonasal can be an effective approach for midline CPs in children (69). The largest adult CP meta-analysis reviewing 22 unique studies providing data for 759 cases with 68.9-month average follow-up, reported recurrence rates among adult CPs of 17% after GTR, 27% after STR + RT, and 45% after STR. The risk of recurrence after GTR vs STR + RT did not reach significance (70).

In their systematic review, Clark et al. found that also in pediatric CPs, there is no difference in 1- or 5-year PFS between the groups who underwent GTR and STR combined with radiation (5-year PFS: 77 vs 73%, respectively) (71).

Hypothalamic damage presents a detrimental impact on long-term co-morbidities, even to a severe degree, potentially impacting long-term survival and quality of life (62, 72). Hypothalamic preservation represents an important goal in driving surgical management and a comprehensive treatment strategy both for adults and children (60, 62, 65).

In patients with hypothalamic involvement, it is generally recommended a treatment strategy based on the combination of maximal resection with hypothalamic sparing and following radiotherapy on residual disease (60, 62, 65).

RT can be delivered as postoperative treatment in cases of residual disease or recurrence, and depending on the surgical strategy based on pre-operative hypothalamic involvement. Many advanced radiation techniques are available nowadays: SRS/hypofractionated stereotactic RT (HFSRT), FSRT, IMRT, and PT. LC rates ranged from 65% to 100%, with the same efficacy expected in terms of LC across different advanced radiation modalities adopted, with a range between different series due mainly to the prevalent retrospective nature of published series and the wide heterogeneity of their populations (73–82).

For example, the presence of cystic disease negatively impacts tumor control. Greenfield et al. for pediatric series with IMRT reported 5- and 10-year cystic disease PFS rates of 70.2% and 65.2%, while the 5- and 10-year solid disease PFS were the same at 90.7% (79).

Bishop et al. for pediatric series treated with IMRT or PT reported that 3-year cystic failure-free survival (CFFS) and nodular failure-free survival (NFFS) rates for the entire group were 75.5% and 95.0%, respectively, with no significant differences for disaggregated analysis for two different techniques (75). The doses adopted were 11–13 Gy in radiosurgical series and 45–54 Gy in conventionally fractionated series (1.8–2 Gy per fraction) (73–82).

The choice of RT dose schedule comes before the choice of radiotherapy modality/technique, as we will discuss widely in the paragraph below on selection criteria for PRT in low-grade skull base tumors. In CPs, the choice of fractionation depends first on the distance of the tumors from the optic pathways (76).

Conventional fractionation is adopted in cases of tumors very close (within 3 mm) or touching/compressing optic pathways, while the radiosurgical/hypofractionated schedule requests at least 3 mm of tumor distance from optic pathways. Furthermore, the larger volume (>3 cm) and complex shape of the tumor, especially in the case of cystic tumors, can influence the choice of conventional fractionation. Regardless of the patients’ age, the PT in patients affected by CP is usually delivered with conventional fractionation of dose (1.8–2 Gy per fraction, 45–30 fractions, total dose 45–54 Gy) (75, 80–82). PT represents an elective option for children and adolescents with brain tumors to spare cognitive function and adaptive performance and ultimately to preserve quality of life (8, 83–93).

Particularly, CPs represent one of the pediatric brain tumor types historically most investigated, related to, and most indicated for treatment with PT (8, 83–85, 93). Dosimetric studies in CPs have shown significant better sparing of healthy tissues, especially brain volumes involved in cognitive performance as hippocampi, comparing PT with photon RT, especially delivered with the IMRT modality (83–85). Furthermore, studies with diffusion tensor imaging (DTI) have been conducted because of its sensitivity to RT-induced alterations in the structural integrity of white matter.

Uh et al. found that in patients with CPs, deep white matter structures developed an early decline during the first year after PT but subsequently recovered, and that surgical defects observed in the corpus callosum before irradiation seemed to prevent complete recovery. These findings can be considered in RT planning to enhance the recovery of white matter (94).

In the second study, the authors found that below-average baseline neurocognitive performance in patients with CPs before PT seems to be related to structural degradation of white matter tracts. Surgery, obstructive hydrocephalus, and preoperative hypothalamic involvement seem to be the main features of these degradations. Longitudinal DTI showed improving trends over 5 years after PT in global integrity and efficiency measures, particularly in children in whom a smaller brain volume was irradiated (95).

In an old clinical series of patients treated with protons, LC rates were 93% at 5 years (96, 97).

Fitzek et al. delivered combined photo-proton RT in 15 patients with a median dose of 56.9 cobalt Gy equivalent (CGE; 1 proton Gy 1⁄4 1.1 CGE) (96). The median PT dose component was 26.9 CGE.

Luu et al. treated 16 patients entirely with PT with a daily dose of 1.8 CGE for a total CGE of 50.4 to 59.4 (97).

In these old-dated series, higher radiation therapeutic doses than those currently adopted and considerations useful for current practice cannot be drawn (96, 97).

Nevertheless, Fitzek et al. reported no treatment-related neurocognitive deficits were recorded within the follow-up period, and functional status, academic skills, and professional abilities were unaltered after PT (96).

In **Table 4**, we summarized selected published series of patients affected by craniopharyngiomas and treated with PT (75, 80–82).

**Table 4:** Patients and treatment description of craniopharyngiomas irradiated with proton radiotherapy (selected series).

Study	Patient characteristics (number and age group)	Follow up (Months/Years)	RT dose (GyRBE)	LC (%)	Late Toxicity
Bishop, 2014 (75)	21 (children, median age 9.1 y)	33 months median	TD: 50.4–54 Dpf: 1.8	3-y NFFS rates: 91.7% 3-y CFFS rates: 67.0%	10% vascular toxicity 5% vision toxicity 19% hypothalamic morbidity 76% endocrinopathy
Ajithkumar, 2018 (82)	16 (13 patients <18 y; median age 10.2 y	32.6 months median	TD: 54 Dpf: 1.8	94%	No high grade toxicity
Rutenberg, 2020 (81)	14 (adults, median age 28 y	29 months median (clinical) 26 months median (radiographic)	TD: 52.2–54 Dpf: 1.8	100% at 3 years	Endocrinopathy G2 (29%) Insomnia G2 (7%) No high grade toxicity No vision loss or Optic neuropathy
Jimenez, 2021 (80)	77 (median age 8.6 y)	4.8 years	TD: 54 (median) Dpf: 1.8	92%	Visual impairment: (no new cases; worsened: 10%) No cognitive changes; moyamoya syndrome: 6% Endocrinopathy (new cases: 7% worsened: 47%)

RT, radiotherapy; TD, total dose; Dpf, dose per fraction; LC, local control; y, years; cystic failure-free survival (CFFS) rates; nodular failure-free survival (NFFS) rates.

Recently, Jimenez et al. published a series on 77 patients affected by CP and treated with PT (from 2002 to 2018) with a median RT dose of 52.2 Gy. Of 77 patients, 76 (97%) received passively scattered protons and 1 (1%) received pencil beam scanning protons, which represent the current standard in PT delivery. The median age at radiation was 9.6 years. The most common presenting symptoms before were headache (58%), visual impairment (55%), and endocrinopathy (40%). Patients underwent a median of two surgical interventions (range, 1–7) before PT. At a median of 4.8 years from RT (range, 0.8–15.6), six local failures were observed, and the 5-year local failure estimate was 9.9%, while the 5-year OS was 97.7%. Only 4% developed acute G3 toxicity. Concerning visual function, the authors observed no patients with new cases of visual impairment after PT. The majority (68%) of patients with pre-existing visual impairments presented stability, with 10% improving and 10% worsening. Among those patients with worsening vision after treatment, six out of the eight patients presented documented pre-treatment poor vision, including three who presented defect severity compatible with blindness. This finding suggests that pre-treatment impairments may make patients more susceptible to additional damage, and specific attention should be focused on these patients to minimize any additional radiation exposure to the optic chiasm and optic nerves. Concerning cognitive function, the Full Scale Intelligence Quotient, Processing Index, and verbal and visual memory scores were stable and did not significantly change. Only adaptive skills showed a statistically significant decrease in mean score at follow-up compared with baseline, but clinically, this decrease was not considered significant as scores remained within the average range for patients. Five patients out of 77 (6%) developed moyamoya syndrome. New endocrinopathies were reported in 7%; among pre-existing cases, they worsened in 47%, were stable in 49%, and improved in 4%. Notably, diabetes insipidus was reduced from 36% pre-PT to 12% post-RT (80).

Bishop et al. reported the outcomes of a clinical comparison study between PT (n = 21) and IMRT (n = 31) in children. The clinical outcomes measured in terms of LC, cyst dynamics after RT, and late toxicity did not show statistically significant differences between the two techniques. Nevertheless, the main parameters testing the critical differences between these radiation modalities were not investigated: the RT dose-volume parameters for supratentorial brain and hippocampi and the neurocognitive outcomes. The 3-year CFFS and NFFS rates for the entire group were 75.5% and 95.0%: 3-year CFFS rates were 67.0% for PT and 76.8% for IMRT, but the biological significance of cyst growth is undefined; 3-year NFFS rates were 91.7% for PT vs. 96.4% for IMRT. The 3-year OS rate was 96% (94.1% PT vs. 96.8% IMRT). The proton cohort presented the following late toxicity profile: 10% of vascular morbidity (versus 10% in the IMRT cohort), 5% of vision morbidity (versus 13% in the IMRT cohort), 19% of hypothalamic obesity (versus 29% in the IMRT cohort), and 76% of endocrinopathy (versus 77% in the IMRT cohort) (75).

In a mixed series with pediatric and adult patients, Ajithkumar et al. reported the early clinical outcomes of 13 children of 16 patients included in a registry study. The control rate was 93%: five patients remained in complete remission, four were in partial remission, and seven had stable disease. There were no treatment-related grade 3 toxicities (82).

Endocrinopathy is a very common clinical morbidity in all patients with CPs, regardless of age, due to disease or treatment. Endocrine outcomes of protons series are comparable with those of other RT techniques considered relevant, with differences in reporting this outcome between different published reports (75, 80).

In the evaluation of the endocrine toxicity rates of the RT series, it should be considered that the pituitary gland is included within the target volume for these tumors. Vascular morbidity and moyamoya syndrome represent a serious concern for the treatment of CPs, especially in children, with 6%–10% rates reported in the above-mentioned proton series (75, 80). Recent studies have reviewed 644 pediatric patients at a single institution to estimate the rate of and identify risk factors for vasculopathy after PT in pediatric patients with central nervous system and skull base tumors (98). The three most common histologies were craniopharyngioma (n = 135), ependymoma (n = 135), and low-grade glioma (n = 131). The authors found the 3-year cumulative rates of any vasculopathy and serious vasculopathy were 6.4% and 2.6%, respectively, and that a maximum dose exceeding or equal to 54 CGE to the optic chiasm was significantly associated with the development of any vasculopathy (13.1% vs 2.2%; P <.001) and serious vasculopathy (3.8% vs 1.7%; P <.05). Interestingly, Lucas et al. found in their phase II prospective study that postsurgical, pre-PT vasculopathy, and PT dose to unperturbed vessels were predictive of vascular stenosis, while the effect of PT on stenosis was negligible within the surgical corridor (99). In the radiation treatment of CPs, the cystic dynamic represents a frequent and critical point in the clinical management of patients during treatment and follow-up. The cyst growth was observed in 13%–40% of cases; adaptive replanning was necessary because cyst growth was beyond the original treatment fields in 12%–24% of cases (75, 80, 100). Bishop et al. observed immediately after RT that 17 patients (33%) had cyst growth (transient in 14) and 27% experienced late cyst growth, with intervention required in 40%. Bishop et al. recommend that if there is asymptomatic early cyst growth immediately after RT, interventions should be avoided and the patients closely monitored. They emphasize the need for close observation and intervention for continued cyst expansion. Bishop et al. reported that cyst growth was related to visual and hypothalamic toxicity (P = 0.009 and 0.04) (75).

Winkfield et al. recommend surveillance imaging be performed at least every 2 weeks during PT to avoid marginal failure, while cases with large cystic components or enlargement during treatment might require weekly imaging (100). Regarding adults affected by CPs, the potential range of applications for PT is more selective. Rutenberg et al. reported in their series of 14 patients with exclusion that 3-year LC and OS rates were 100%. There were no G3 or greater acute or late radiotherapy-related side effects. There was no RT-related vision loss or optic neuropathy. No patients required intervention or treatment replanning due to tumor changes during RT (81). Ajithkumar et al. reported a series of 16 CPs treated with PT, on which three were adults. The patients received 54 Gy (RBE) of PT and a follow-up of 25 months. The three patients exhibited a complete radiological response (82).

In adult patients, the PT finds its potential application in patients not suitable for SRS and requiring conventional fractionation for larger/giant tumor volumes (maximum diameter >3 cm) and tumors with proximity to or involvement (abutting/compression) of optic pathways. The PT should be chosen among available high-precision radiotherapy modalities (such as intensity modulated techniques and fractionated stereotactic radiotherapy) that permit treatment with conventional dose fractionation when this schedule should be preferred, as discussed widely in the paragraph dedicated to clinical selection criteria for PT in skull base low-grade tumors.

## Pituitary adenomas

Pituitary adenomas are usually low-grade tumors but have a significant impact on the daily quality of life of patients and health care systems. Increased availability of MRI has resulted in an increase in incidentally found pituitary lesions and clinically relevant pituitary adenomas.

Epidemiologic studies show that pituitary adenomas are increasing in incidence (between 3.9 and 7.4 cases per 100,000 per year) and prevalence (76 to 116 cases per 100,000 population) in the general population (approximately one case per 1,000 of the general population) (101).

Approximately 50% are microadenomas (<10 mm); the remaining are macroadenomas (≥10 mm) and giant adenomas (≥40 mm). Pituitary carcinomas with distant metastases are rare, occurring in 0.1% to 0.2% of cases. About two-thirds of pituitary adenomas may secrete excess hormones (102).

In 2022, the 5th Edition of the WHO Classification of Endocrine and Neuroendocrine Tumors was published. Regarding the section dedicated to the pituitary gland, the new classification clearly distinguishes the anterior lobe (adeno-hypophysis) from posterior lobe (neuro-hypophysis) and hypothalamic tumors. In the anterior lobe, tumors were well-differentiated adenohypophyseal tumors that are now classified as pituitary neuroendocrine tumors (PitNETs; formerly known as pituitary adenomas). The routine use of immunohistochemistry for pituitary transcription factors (PIT1, TPIT, SF1, GATA3, and ERα) is included in this classification. The major PIT1, TPIT, and SF1 lineage-defined PitNET types and subtypes have distinct morphologic, molecular, and clinical differences. The “null cell” tumor, which is a diagnosis of exclusion, is reserved for PitNETs with no evidence of adenohypophyseal lineage differentiation. The term “metastatic PitNET” is advocated to replace the previous terminology of “pituitary carcinoma” (103). The treatment options include trans-sphenoidal surgery, medical therapies, and radiotherapy. Trans-sphenoidal surgical resection of adenomas represents the initial treatment option for all tumors except prolactinomas, for which medical therapy represents the first-line option (103).

Endoscopic transsphenoidal surgery in comparison with microscopic trans-sphenoidal surgery is associated with a higher GTR, no significant effect on the risk of cerebrospinal fluid leak, a reduction in the risk of diabetes insipidus, and a significantly reduced risk of septal perforation (104). SRS represents an effective treatment option regardless of the fractionation dose schedule adopted for both non-functioning and secreting pituitaries with residual or recurrent disease post-surgery or with refractory disease after medical therapy (105). Based on available data, there is no evidence supporting the superiority of SRS over FSRT for the treatment of patients with pituitary adenomas. The dose and fractionation schedules are usually prescribed based on the size and position of the pituitary adenomas (105). The single-fraction SRS may represent an appropriate approach for patients with small and medium-sized pituitary adenomas far at least 2 mm from the optic chiasm or optic nerves, while FSRT is more indicated over SRS for lesions >2.5–3 cm in size and/or involving optic pathways (105).

Some series suggest that multi-fraction SRS may be an adequate option in patients with tumors in proximity to the optic apparatus (106–109).

Recent systematic reviews and meta-analyses regarding the role of stereotactic radiosurgery in pituitary adenomas have been published by the International Stereotactic Radiosurgery Society (110, 111).

In SRS for non-functioning tumors, the following results have been reported (110). The 5-year random effects LC estimate after SRS was 94% and 97.0% after HSRT. The 10-year local control random effects estimate after SRS was 83.0%. Post-SRS hypopituitarism was the most common treatment-related toxicity observed, with a random effect estimate of 21.0%, while visual dysfunction or other cranial nerve injuries were uncommon (range: 0%–7%). The authors recommended a prescription dose of 14–16 Gy for patients treated in the definitive setting and patients with residual or recurrent disease. Hypofractionated RT (21 Gy in three fractions, 20 Gy in four fractions, or 25 Gy in five fractions) can be considered for patients with larger adenomas (>2–3 cm) or close to the optic apparatus, but it should be carefully considered due to the acknowledged lack of long-term tumor control data (110).

In SRS for secreting tumors, the following results have been reported in a systematic review published by Mathieu et al. (111).

Random effects meta-analysis estimates for crude tumor control rate, crude endocrine remission rate, and any new hypopituitarism rates ranged 92%–97%, 28%–48%, and 12–21%, respectively (111). Mean margin doses reported ranged from 13.2% to 35%, and new neurological or visual deficit rates ranged between 0% and 17%. No minimal margin dose was shown to definitively lead to better endocrine cure rates (111). Several authors supported the use of doses >30–40 Gy (112–114).

Mathieu et al. recommend that higher margin doses can be used, provided dose constraints safely protect surrounding structures at risk (optic pathways, brainstem) (111).

The FSRT achieves local control rates ranging from 91 to 100% and prescribed doses range from 45 to 54 Gy (1.8–2 Gy/fractions). Visual toxicity ranged from 0 to 7.5% and hypopituitarism ranged from 3 to 48% (105).

The published PT series included three series adopting protons with radiosurgical schedules (115–117) and one series treating patients exclusively with conventional fractionation schedules (118).

In **Table 5**, we summarized a selected published series of patients affected by pituitary adenomas and treated with PT (115–118).

**Table 5:** Patients and treatment description of pituitary adenomas irradiated with proton radiotherapy (selected series).

Study	Patient characteristics (number and age group)	Follow up (Months/Years)	RT dose schedule (GyRBE)	Disease Control	Late Toxicity
Ronson, 2006 (118)	47 (51% no functional) -	47 mo.	PFRT: TD: 54 median Dpf: 1.8–2	**Whole series:** Complete Tumor Regression: 24.4% Partial Tumor Regression: 29.3% Tumor Stabilization: 46.3% **Functional adenomas:** biochemical control: 85.7% CR: 38.1% PR: 47.6%	No visual worsening: 76.7% Minor visual complications: 23% Major visual complications: 4.6% New pituitary defect: 29.7% Developed panhypopituitarism: 5.4% Brain injury: one patient
Petit 2007 (116)	22 with acromegaly	6.3 y Median	**PRS** TD: 20 Dpf: 20	CR rates: 59%	No evidence of visual complications, seizure, or brain injury New pituitary deficit: 38%
Petit, 2008 (115)	38 patients 33 (CD) (range 19–60 y) 5 (NS) (range 29–53 y)	62 mo. Median	**PRS** TD: 20 Dpf: 20	(CD): CR rates: 52% 5-y CR: 55% (NS): CR rates: 100%	No evidence of optic nerve damage, seizure, or brain injury New pituitary deficit: 53%
Wattson, 2014 (117)	165 mixed Functional adenomas	4.3 y median	PRS (92%): TD: 20 Dpf: 20 PFRT (8%): TD: 50.4 Dpf: 1.8	5-y CR: 59% LC: 98%	5-y new pituitary deficit: 62% Seizures: 2% (4/165)

RT, radiotherapy; PRS, Proton Radiosurgery; PFRT, Proton Fractionated Radiotherapy; TD, total dose; Dpf, dose per fraction; LC, local control; y, years; mo, months; CR, Complete Response (biochemical); PR, Partial Response (biochemical); CD, Cushing’s disease; NS, Nelson’s syndrome.

The proton radiosurgical series adopted a dose of 20 Gy (RBE) and obtained the following results in terms of disease control outcomes. Wattson et al. reported in 165 mixed functional adenomas 98% of local control and 59% of 5-year hormonal normalization rates (117). Petit reported that in 38 ACTH-secreting adenomas, complete remission (CR) occurred in 52% of patients with Cushing disease, and the median time to complete remission was 14 months (range 5–49). Actuarial rates of CR at 5 years were 55%. Complete remission was obtained in all patients with Nelson’s disease (115). Petit reported in 22 patients, with an acromegaly complete response achieved in 13 patients (59%). Among patients with CR, the median time to CR was 42 (range, 6–62) months (116).

In these series, new pituitary deficits were reported in the range of 38%–62% (115–117).

Ronson et al. reported outcomes of fractionated PT with 54 Gy CGE for treatment of pituitary adenomas: 100% of LC with 29.3% partial tumor regression and 24.4% complete tumor regression at last follow-up, while 85.7% had normalized or decreased hormone levels at last follow-up. New hypopituitarism cases were observed in 11 patients. In this series, seven patients developed minor visual deficits, and two patients developed major visual deficits that consisted of a new quadrantanopsia and bilateral optic nerve atrophy. Both patients had Cushing’s disease, and the authors hypothesize that the long-term effects of hypercortisolism make the optic chiasm microvasculature of Cushing’s patients susceptible to radiation injury. Furthermore, dose fractionation permits treatment of larger target volumes that may be adjacent to or even compress the optic pathways,resulting in a higher risk of injury. In these patients, particular caution is requested in radiation treatment planning, especially if additional risk factors for radiation damage are present (118).

The radiosurgical dose schedule can usually be indicated when the tumor presents at least 3 mm of distance from the optic pathways, and this condition favors fewer visual complications. In the most challenging cases of patients with optic pathways directly involved by tumors with an abutment or compression pattern, the conventional fractionated schedule is more indicated to preserve residual visual function and minimize visual worsening as much as possible. In these situations, the visual function is often variously compromised by previous radiotherapy, and this condition subjectively enhances the susceptibility to radio-induced visual worsening. For these biased pre-treatment conditions in radiation treatment delivered with conventional fractionation, we can observe more cases of visual complications. Generally, in published series, minimal neurological toxicity has been recorded (115–118).

Nowadays, the PT in the treatment of pituitary adenomas can find a real perspective of clinical application when a conventional fractionated dose schedule is required: in cases with larger-sized/giant tumor volumes and cases with tumor in very close proximity or involving optic pathways by abutting or compressing. The issue of selection criteria for low-grade skull base tumors is discussed in a dedicated paragraph.

## Vestibular schwannomas

Vestibular schwannomas (VS, formerly termed acoustic neuromas) are usually benign tumors derived from Schwann cells. VS develops from the nerve sheath of the vestibular division of the vestibulocochlear nerve (VIII cranial nerve) at the internal auditory meatus (119).

VSs represent the third most common intracranial non-malignant tumor entity**;** incidence rates range between 1.1 and 1.9 per 100,000, and usually a diagnosis is made in the third to fifth decades of life (120–122).

The majority of VSs occur unilaterally and sporadically. The most well-documented risk factor for VS development is NF2, 4%–6% of VS are associated with NF2. These patients typically develop bilateral disease as well as multiple other tumors (120, 121).

The diagnosis is made with contrast-enhanced magnetic resonance imaging. Patients often present with unilateral sensorineural hearing loss, tinnitus, and vertigo with gait disorders. Large tumors may cause neuropathies (trigeminal and facial nerves) as well as brainstem compression and hydrocephalus (120).

Furthermore, VS may be found incidentally on imaging examinations; the increased use of MRI imaging has also led to an increase in the diagnosis of smaller VSs.

Koos grading scale (KGS) (120, 122, 123) is a score frequently used for VS. KGS is designed to stratify tumors and is divided into four grades based on extra-meatal extension and compression of the brainstem:

– Grade I = small intracanalicular tumor.
– Grade II = small tumor with protrusion into the cerebello-pontine angle (CPA); no contact with the brainstem.
– Grade III = tumor occupying the cerebellopontine cistern with no brainstem displacement.
– Grade IV = large tumor with brainstem and cranial nerve displacement.

Standard management of VS includes observation, surgery, SRS, or conventional RT (120). The type of treatment is typically based on tumor size and its impact on adjacent brain structures (120, 122, 123).

Due to the slow progression of VS, the “watch and wait strategy” can be a legitimate treatment option for selected patients (120).

Surgical management of VS should be based on tumor size and morphology, symptoms, comorbidities, and patient preferences (120, 122, 123). Surgical resection offers excellent local tumor control but has been associated with a significant risk of injury to the V, VII, and VIII cranial nerves. In VS of Koos grade IV, surgery should be the primary treatment to remove a symptomatic lesion or potentially life-threatening mass effect (120). Surgery may also be considered for smaller tumors with cystic degeneration or if the cure is the primary goal of treatment (120).

RT can be delivered through several modalities, including SRS, which uses a single high-dose fraction, and conventionally fractionated RT (FRT), which uses smaller daily doses typically delivered in 28 to 32 fractions (119, 120, 124–128).

SRS defines the delivery of high-dose irradiation with high conformity and precision in a single fraction and is commonly used for small to medium-sized VSs. SRS can be performed using GammaKnife or CyberKnife at doses ranging from 11 to 14 Gy (120, 128).

SRS is used as a noninvasive approach for definitive treatment of small to medium-sized or recurrent tumors as it offers excellent rates of local control and better functional outcome and quality of life (QOL) compared to surgery (119, 120, 128).

SRS and FRT with modern techniques can achieve similar results in terms of local control and hearing function, although FRT can be used when surgery is not feasible or when a patient has larger VSs (Koos grades 3–4) in close proximity to the brainstem (119, 120, 127, 128).

Fractionated proton radiotherapy (FPT) for VS achieves high tumor control rates, equivalent to photon FRT techniques. Furthermore, FPT has peculiar physical properties that allow it to give more radiation energy to the target, sparing the surrounding normal tissues and thus having the potential to reduce treatment-associated toxicities (120, 124, 125, 127, 128). To date, there are few data points on PT for VSs.

In **Table 6**, we summarize selected published series of patients affected by pituitary adenomas and treated with PT (119, 124–127).

**Table 6:** Patients and treatment description of vestibular schwannomas irradiated with proton radiotherapy (selected series).

Study	Patient characteristics (number and age groups)	Follow up (Month)	RT dose (GyRBE)	LC (%)	Late Toxicity
Weber, 2003 (126)	88 (median age 69.2y)	38.7	Median prescribed dose: 12 CGE (10–18) Median maximal tumor dose: 17.1 CGE (13.3–20) Isodose line percentage prescription: 70% (70–108) No. of fractions: 3 (2–4)	2-y: 95.3% 5-y: 93.6%	Permanent facial nerve dysfunction (HB Grade 3–4: 4 patients) Permanent “significant” trigeminal nerve dysfunction: two patients
Barnes, 2018 (125)	95 (median age 56 y) 43 (Group: 50.4 Gy) 34 (Group: 54.0 Gy) 19 (Group: 59.4 Gy)	4.3 y (Group: 50.4 Gy) 7.4 y (Group: 54.0 Gy) 6.6 y (Group: 59.4 Gy)	TD: Group: 50.4 Gy Group: 54.0 Gy Group: 59.4 Gy Dpf: 1.8 Gy	5-y LC: 92% (Group: 50.4 Gy) 95% (Group: 54.0 Gy) 97% (Group: 59.4 Gy) Overall 10-y: 90%.	No high grade toxicity
Zhu, 2018 (124)	14 (median age 60 y)	68	TD: 50.4 Gy Dpf: 1.8 Gy	3-y: 85%.	No high grade toxicity
Eichkorn, 2021 (127)	45 (median age 55 y)	42	TD: 54 Gy Dpf: 1.8 Gy	100%	No high grade toxicity. Radiation-induced contrast enhancements (seven patients,16%): G1–G2
Küchler, 2022 (119)	261	38	SRS/HFSRT (TD:12 Gy, single fraction; TD 18 Gy, Dpf 6 Gy) FRT (TD: 57.6 Gy; Dpf: 1.8 Gy) FPT (TD: 54GyRBE), Dpf: 1.8/GyRBE).	1 y: 99.5%; 3 y: 93.7%; 6y: 90.8%; No statistical difference between treatment groups (p = 0.19)	No high grade toxicity.

RT, radiotherapy; CGE, cobalt Gray equivalents; TD, total dose; Dpf, dose per fraction; LC, local control; y, years; HB, House-Brackmann; SRS/HFSRT, stereotactic radiosurgery/hypofractionated stereotactic radiotherapy; FRT, fractionated radiotherapy; FPT, fractionated proton therapy.

A retrospective cohort study investigated proton-beam stereotactic radiosurgery for VS. It was reported that there was a 5-year tumor control rate of 93.6% and a dose dependency for facial neuropathy (126).

Barnes et al. prospectively investigated efficacy and toxicity rates in 94 patients who underwent FPT for VS. FPT at a daily dose of 1.8 Gy (RBE) was employed (125). Patients were treated with one of three total dose options: 59.4 Gy (RBE), 54 Gy (RBE), or 50.4 Gy (RBE). Five-year local control rates for the 59.4 Gy (RBE), 54 Gy (RBE), and 50.4 Gy (RBE) groups were 95%, 97%, and 92%, respectively; the overall 10-year control rate was 90%. These data demonstrated a dose-dependent risk for hearing deterioration of 36% to 56% at doses from 50.4 to 54 Gy (RBE), while the risk for damage to other cranial nerves was 5%. FPT of 50.4 Gy (RBE) offers excellent LC rates with minimal cranial nerve toxicities (125).

Zhu and colleagues reported a retrospective case series of 14 patients who received 50.4 Gy (RBE) in 28 fractions of 1.8 Gy (RBE)/fraction (124). The 3-year LC rate was 85%, with no cranial nerve V or VII injuries. Twenty-one percent of patients had a radiographic tumor regression on MRI after a median of 26 months. No acute toxicity of G3 or above was reported.

Eichkorn et al. analyzed 45 patients who underwent FPT with a median total dose of 54 Gy (RBE) at 1.8 Gy (RBE)/fraction (127). It was reported that there was 100% local control in a median follow-up period of 3.6 years, and MRI revealed 93.3% of stable disease and 6.7% of partial regression. There was no case of progressive disease. New or worsening cranial nerve dysfunction (G1–2) was found in 20.0% of all patients. In 16% of cases, radiation-induced contrast enhancements (RICEs) were detected after a median of 14 months. RICEs were asymptomatic (71%) or transiently symptomatic (G2; 29%). No G3 or G4 toxicities were observed.

Küchler and colleagues reported a retrospective exploratory analysis to evaluate differences in tumor control, symptoms, and quality of life in VS patients after SRS/HFSRT, FRT, and FPT (119). For SRS/HFSRT, the median fraction dose applied was 12 Gy. For FRT and FPT, the median doses applied were 57.6 Gy and 54 Gy (RBE), respectively. FRT and FPT used single median doses of 1.8 Gy (RBE). Local control was 99.5% at 12 months after RT, with no statistical difference between treatment groups. SRS/HFSRT, FRT, and FPT for VS show similar functional outcomes. Cranial nerve impairment rates vary, potentially due to selection bias with larger VS in the FRT and FPT groups (119).

The hearing preservation rate varies among these cases because of the heterogeneity of treatments administered. Weber et al. evaluated proton-beam stereotactic radiosurgery with a hearing preservation rate of 79.1% and 21.9% at the 2 and 5-year follow-up, respectively (126).

In Barnes et al., 43% of 54-Gy-group patients maintained functional hearing during a median follow-up time of 58.2 months, with a median time to onset of the unserviceable hearing status of 14.8 months. Instead, 64% of the 50.4-Gy group maintained functional hearing with a median follow-up time of 42.7 months. The median time to hearing loss in patients who did not preserve useful hearing was 12 months. The difference in serviceable hearing between the 50.4-Gy and 54-Gy groups at 24 months and 48 months was not statistically significant (125).

Zhu et al. described the outcome of conventional FPT for VSs: the retained serviceable hearing in patients with baseline serviceable hearing was 33% (two patients) with a median follow-up of 70 months (124). Eichkorn et al. did not highlight acute or late hearing loss after PT (127). Küchler et al. reported a hearing preservation rate of 97.1%, 94.2%, and 87.1% at 12, 24, and 60 months in patients with useful hearing before treatment. Hearing deterioration was underlined in 17.8% and 16.7% of patients treated with fractionated radiotherapy (photon and PT, respectively) and in 3.6% of patients treated with SRS/HFSRT (119).

Regarding identifying patients affected by VS, cases requiring conventional FRT in an elective way can potentially be indicated for particle radiotherapy (and especially proton radiotherapy), as discussed widely in a dedicated paragraph regarding selection criteria for particle therapy in low-grade skull base tumors.

## Low grade skull base tumors: considerations on selection criteria for PRT

Low-grade skull base tumors include WHO-G1 meningiomas (or presumed WHO-G1 in cases with an exclusive radiological diagnosis), craniopharyngiomas, pituitary adenomas, and vestibular schwannomas. For these types of tumors, lower levels of effective radiation doses are required. Consequently, high local control rates and the same efficacy obtained by PRT in comparison with advanced photon RT techniques are expected for these low-grade tumor types.

The selection criteria for PRT compared with photon radiotherapy are mainly based on the evaluation of the achievement of the goal represented by toxicity minimization and functional preservation.

Many advanced modalities and techniques are available for photon RT (SRS techniques and IMRT modalities), and for PT, the delivery techniques have been refined, evolving from passive scattering to active scanning technology, which represents the current standard for PRT (51, 52, 54, 80–82, 105, 128–131).

Among PRT options, carbon ions are not suitable as primary radiation treatment for patients affected by low-grade tumor types due to their high RBE and consequent overtreatment in terms of therapeutic ratio, and only PT is indicated in this subset of patients.

When recurrent previously irradiated low-grade tumors switch towards more aggressive biological behavior or a higher histology grade, CIRT can be re-considered, especially if valid and effective therapeutic alternatives are not available. In these cases, we are faced with *de facto* radioresistance, regardless of histological type, and CIRT is particularly indicated in cases of radioresistant tumors (56, 132, 133).

As previously introduced, in the treatment of low-grade skull base tumors, considering the wide availability of precise RT options and the lower effective radiation dose required, the same high probability of disease control and low toxicity rates are reasonably expected with either proton or photon RT advanced techniques and the same treatment volume identification criteria across RT techniques, are adopted (51, 52, 54, 73–75, 80, 81, 105, 115–119, 128–130, 134).

A competitive approach between different radiation technical modalities does not help in the choice of a better radiation option. In making the decision-process of each clinical case, the evaluation and choice of the most suitable fractionation radiation dose schedule based on tumor volume and spatial relationship with the organ at risk represents the first step, and the choice of the radiation modality and technique represents a secondary step (54, 76, 105, 119, 120, 127–130, 135, 136).

Regardless of tumor histology, low-grade skull base tumors with small-medium size or at most larger volumes with a maximum diameter of 3–3.5 cm and at least 3 mm distance from the brainstem and optic pathways are typically suitable cases for radiosurgical schedules (up to a multi-session schedule with five fractions) with different technology delivery options (GammaKnife, CyberKnife, or other LINAC machines with radiosurgical equipment, proton radiosurgery) (76, 105, 115–117, 119, 120, 128, 129, 135, 136).

In several cases, a conventional fractionation schedule (1.8–2 Gy/fraction) is more indicated in skull base low-grade tumors considering the critical location represented by the skull base: very large or giant tumors; tumors closely involving the brainstem and/or optic pathways by abutting, compressing and enveloping these structures. In these cases, conventional fractionation has been well recognized as preferable to minimize toxicity in the brainstem, optic pathways, and other cranial nerves (54, 74, 76, 81, 105, 118–120, 127–131, 135–140).

The IMRT techniques (tomotherapy, VMAT) represent a photon RT option for very large/giant, and complex-shaped tumors with conventional fractionation, but compared with this option, the PT can more effectively spare neurocognitive function by minimizing dose delivered to hippocampi and brain (84, 85, 141).

Especially in patients with low-grade tumors and a favorable long-term prognosis, PT significantly reduces the risk of radio-induced malignancy (142, 143).

The sparing of neuro-cognitive function represents a major concern in the irradiation of intracranial tumors, both in children and adults. Neurocognitive impairment negatively affects the quality of life and instrumental activities of daily living (86, 144, 145).

The cause of neurocognitive decline in patients with intracranial tumors is multifactorial. The further RT, several factors are associated with impairment in neurocognitive factors: the tumor itself and its features (size, location, type, and grade; initial versus recurrent disease); medical treatment as corticosteroids and anticonvulsants; metabolic/endocrine dysfunction; the impact of surgery; the number of surgeries; the ventriculoperitoneal shunt; postoperative complications; and many others. The extent of the contribution of radiotherapy relative to other factors is not known or quantifiable. Multiple pathophysiological mechanisms have been suggested to explain the brain injuries and consequent cognitive impairment induced by RT, including impairment of neurogenesis (145–150).

Hippocampi represent a relevant region for neurocognitive function outcomes, but several other regions in the brain and various healthy cerebral tissues are potentially involved in the pathophysiology of cognitive impairment, as, for example, cerebral white matter, cerebral cortex, and subventricular zones (145, 146, 151, 152).

The impact of several RT dose parameters on neurocognitive function reported in the literature supports the idea that reduction of radiation dose–volume relationships between hippocampi and brain volume can positively affect the preservation of neurocognitive functions. Particularly considering the important role of the hippocampus in terms of cognitive function, hippocampal-sparing approaches in cranial radiation treatment have been developed in recent years (87, 88, 145, 146, 151–159).

As above-mentioned, PT permits significantly reduced radiation dose-volume delivery to the brain volume and hippocampi, and consequently, this dosimetric advantage can determine better outcomes in terms of neurocognitive sparing, as supported also by normal tissue complication probability (NTCP) modeling studies (84–90, 141, 160–164).

In children, adolescents, and young adults, the role of PT as an elective radiation modality, especially in brain tumors, has been increasingly supported in recent years (8, 83–93).

Summarizing, the PT could represent the elective radiation modality for patients affected by low-grade skull base tumors with indications for conventionally fractionated radiotherapy and cases not suitable for a radiosurgical schedule: larger-sized and/or complex-shaped tumors; tumors with proximity and involving brainstem and/or optic pathways. Furthermore, PT should be considered the first option among radiation modalities for these patients.

## Conclusions

PRT represents an effective and safe therapeutic option for skull base tumors.

In radioresistant skull base tumors such as chordomas and sarcomas, the PRT permits higher dose levels required with optimal dose coverage and higher local control probability while minimizing dose to the critical organs and toxicity.

Furthermore, in radioresistant tumor types and recurrent tumors previously irradiated, carbon ions have an intrinsic and peculiarly higher RBE and are more capable of overcoming radioresistance compared with protons, regardless of features such as hypoxia, cell phases, and dose schedule fractionation.

Due to the prognostic factors affecting disease control, in these tumors, the combination of maximally safe surgical resection and high-dose PRT should be the goal of the treatment strategy.

In cases not amenable to attempted gross total/near total removal, the combination of debulking surgery providing space between tumor and brainstem and/or optic pathways followed by PRT should be evaluated.

Considering the critical location represented by the skull base, these patients should be referred to highly specialized centers for skull base surgery.

Furthermore, close and continuous cooperation between surgeons and particle radiation oncologists should favor the planning of a shared optimal combined treatment strategy.

In the skull base location of low-grade tumors, protons are the particle currently adopted.

In low-grade tumors, patients are potentially eligible for PT when the cases require a preferentially conventionally fractionated dose schedule, and if they are not amenable to SRS/HFSRT:

a) tumors with very close proximity or direct involvement (compression or abutment) of the brainstem and/or optic pathways
b) larger, giant sized, and/or very complex-shaped tumors.

Primarily, the dose sparing of brain volumes and subvolumes, such as hippocampi, is correlated with neuro-cognitive function, as well as the minimization of secondary tumor risk, strongly supporting the use of PT when fractionated radiation dose is preferentially indicated in comparison with IMRT techniques.

Even in high-grade meningiomas, a conventional fractionation radiation schedule is required as recommended in EANO guidelines (39) and the above-mentioned considerations for low-grade tumors have amplified implications considering the higher dose required in these tumor types (44, 165). Regardless of skull base location, systematic reviews support the role of PRT in terms of efficacy, local control, and survival rates in high-grade meningiomas, considering that PRT allows for more targeted treatment plans that may limit excess radiation damage for tumors generally considered difficult to manage (166, 167).

In children, adolescents, and young adults, PT should be the preferred option, when available, for the radiation treatment of low-grade skull base tumors.

Considering the critical location of skull base tumors, high-quality and advanced pretreatment MRI imaging and a careful, highly detailed, and comprehensive evaluation in the process of treatment target volume delineation are closely required and represent a crucial point to perform an optimal and high-quality assured radiation treatment (134).

Multi-institutional and collaborative efforts will be important to further increase our knowledge of the management and treatment of skull base tumors.

## Author contributions

All authors listed have made a substantial, direct, and intellectual contribution to the work and approved it for publication. All authors have read and approved the final manuscript.
